# Predicting food waste in households with children: socio-economic and food-related behavior factors

**DOI:** 10.3389/fnut.2023.1249310

**Published:** 2023-10-09

**Authors:** Pietro Tonini, Pere Muñoz Odina, Xavier Gabarrell Durany

**Affiliations:** ^1^Sostenipra Research Group (2017 SGR 1683), Unitat de excelencia Maria de Maetzu MDM CEX2019-000940-M, Institut de Ciència i Tecnologia Ambientals (ICTA-UAB), Universitat Autònoma de Barcelona, Barcelona, Spain; ^2^Department of Chemical, Biological and Environmental Engineering, School of Engineering, Universitat Autònoma de Barcelona (UAB), Barcelona, Spain

**Keywords:** waste, household, parametric model, food related behavior, socio-economic

## Abstract

**Introduction:**

The consumption phase accounts for approximately half of the food waste generated within the food system. Numerous studies have identified families with children as the primary contributors to food waste. The aims of this paper is to enhance the comprehension of food waste behaviors in households with children by characterizing it and studying how socioeconomic characteristics and food-related behaviors can predict it.

**Methods:**

A survey was conducted among 806 families with children, categorized by the child’s age and family structure. The study utilized descriptive statistics to summarize the food waste behaviors and binary regression to evaluate the predictive abilities of 12 variable related to the socio-economic characteristic, purchase, and preparation behaviors and diet quality factors.

**Results:**

Perishable food items, such as fruits, vegetables, cereal-based product, and dairy products, were the primary items wasted in households with children. Two patterns of food waste were identified: inadequate food management leading to small amounts of waste in families with young and middle-aged children, and over-purchasing perishable items leading to waste in other households with children. Household type and purchasing habits were significant predictors, while the purchaser’s age and buying channel showed lower predictive capacity.

**Discussion:**

Policies to reduce food waste should prioritize raising awareness among children, promoting good practices at the household level, and creating favorable conditions during purchases. Strategies include enlisting children’s participation in meal planning and food preparation as well as limiting the promotion of ultra-processed products and incentivizing the sale of bulk products at supermarket.

## Highlights

- Perishable foods and dairy product are the most important contributors of food waste;
- Two distinct patterns of food waste identified in households with children;
- Household type and shopping list use as key factors in predicting food waste generation;
- Policies should focus on young families with small and medium-sized children;

## 1. Introduction

The exponential growth of the global population by 2050 is expected to drive a 50% increase in demand for food products, thereby intensifying resource consumption and greenhouse gas emissions associated with the food system (1, 2). Therefore, it is crucial to increase resource use efficiency to prevent food insecurity and reduce the food system’s environmental impact in the foreseeable future. Among the strategies, the reduction of food waste has been identified as a practical measure to improve resource use efficiency and increase food availability while reducing pressure on natural resources (3). In particular, the consumption stage of the food value chain has received increasing attention, as it generates the highest contribution in terms of quantities and environmental impacts (4, 5). Consequently, reducing domestic food waste has become a crucial global target under Sustainable Development Goal 12.3 (6), particularly in high-income regions such as the United States of America and Europe, where the problem is particularly acute (7).

Domestic food waste comprises spoilage, products thrown away without use, and leftover meals (8). Food spoilage constitutes the bulk of food waste and is easy to estimate since the entire product is discarded; meanwhile, leftovers are the least quantifiable form of food waste among consumers (9). The composition of household waste primarily comprises items that are associated with high levels of consumption and low economic value (10). Specifically, perishable items such as fruits, vegetables, and baked goods are responsible for generating more than half of the food waste produced by households, whereas animal-based products such as meat, fish, and eggs contribute to a negligible proportion of the waste stream [(11, 12)].

Many authors revealed that households with children tend to generate more food waste than households without children (13–17). In particular, research has demonstrated that the quantity of food waste increases as the number of children in the household increases, and the drivers of food waste may vary based on the child’s age (18). Households with young children and adolescents commonly over-purchase and over-prepare food due to their changing food preferences (15), picky eating behaviors (19), and parental intention to provide nutritious and suitable meals for their children (20). In contrast, in households with older children, the primary reason for food waste is the difficulty in predicting their eating habits, such as their presence during mealtimes (21).

Despite this evidence, there still needs to be more representative, reliable primary data on food waste generation at the household with children level (22). In particular, more empirical studies are required to unveil the drivers of food waste and existing framework conditions leading to domestic food waste in these household (23). This segmentation is required to pave the way to curb domestic food waste and point out directions to design policies and interventions specifically for this type of household (24). Indeed, interventions targeting specific characteristics of homogeneous groups of consumers have been proven to be more effective than “one-size-fits-all” ones (25).

The aim of this paper is to enhance the comprehension of food waste behaviors in households with children by characterizing it and studying how socioeconomic characteristics and food-related behaviors can predict it. A questionnaire was administered to 806 families with children categorized by life-cycle stages based on the child’s age and family composition. Unlike previous questionnaire-based studies (13, 15, 26), the objective of this research was not to measure the amount of food waste produced at home but rather to determine which households with children were more likely to produce food waste at home. A binary regression model was used to assess the predicting capacity of the socioeconomic factors and food-related behaviors in the household with children. The variable included in the model were identified in studies with the same focus but with different sample designs (13, 15, 20, 27, 28). The results were used to identify interventions and policy implications that can target specific aspects of drivers to influence food waste behaviors.

## 2. Methods

### 2.1. Study design

The current investigation is part of an exploratory analysis that investigates household expenses, food waste behavior, and residue generation in the population of Cerdanyola del Valles, a municipality situated in the Vallès Occidental region of Catalunya, Spain, with an estimated population of 60,000 people (29). Three online surveys with different designs and objectives were conducted during the study, involving 1,854 households in the municipality. Our study administered the questionnaire to 806 households, with 23% responding to at least one of the two preceding surveys. The questionnaire collected data on food waste behaviors, socioeconomic characteristics, food purchase and preparation behaviors, and diet quality. A professional market research organization was contracted for the recruitment and data collection of the survey. The sample was selected based on the percentage of households with young children (<5 years old) (27%), households with middle age children (5–17 years old)(36%), households with mayor age children (18–30 years old)(25%) and single-parent households (mother or father, children<30 years old)(12%) present in the Spanish population with representative quotas for gender and age (Sampling error: 3.8%) (30). For further details on the socio-demographic characteristic lifecycle stage see also Supplementary Table S1 in the Supplementary Material.

Only the person responsible for at least half of the shopping trips and preparing meals at home were involved in the study. The survey encompassed a total of 14 questions that were administered in both the Catalan and Spanish languages. The initial segment of the questionnaire centered on food waste behaviors, while the subsequent segment inquired into socioeconomic attributes and food-related behaviors. The survey was conducted online and presential between October 2022 with CAWI methodology, for a total of 15 min for each interview. The in-person interview was conducted in supermarkets, municipal markets, and grocery shops to obtain a heterogeneous sample. Before administering the questionnaire, a pre-test was carried out with 25 families with children to check the comprehensibility of the questions and answers and to calculate the average interview time.

### 2.2. Ethical statement

The Research Ethics Committee of the Autonoma University of Barcelona (Barcelona, Spain) approved this study protocol (code number 5539). All procedures were in accordance with the ethical standards established by the Declaration of Helsinki (31). All respondents were informed about the objectives and procedures of the study and provided informed consent before filling out the survey, which was compliant with the General Data Protection Regulation (GDPR). The processing of personal data complies with current Spanish and European legal regulations on data protection.

### 2.3. Food waste behavior

Respondents were asked whether they had discarded edible food in the past seven days to investigate food waste behavior. Food waste was defined as any edible food, liquid or solid, not intended for human consumption, including disposal in the garbage bin, use as pet food, or composting organic matter, as defined by the High-Level Panel of Experts on Food Security and Nutrition (32). Therefore, the study excluded the inedible portion of the products to identify only the household that discarded food which could have been consumed. A seven-day recall period for the food waste question was intended to simplify responses and reduce potential respondent bias (33).

In order to characterize food waste, the person responsible for purchasing food was asked to list the products or dishes that had been discarded during the week. The questionnaire’s administrator subsequently categorized the product or dish into food product categories, such as fresh vegetables, fresh fruit, fresh bread, cereal products, milk and dairy products, animal-based products (e.g., fish, meat, and eggs), frozen or canned products, and ready-to-eat or ready-to-made products. If a dish was composed of multiple products, only the category of the main ingredient was recorded in the questionnaire. Additionally, the disposal reason of the product category was recorded to classify them in quantity-related problems at purchase, quantity-related problems at home and durability. The quantity-related problems are related to the purchase of a too large packaging size or wrong product as well as the preparation of too much food at home. Meanwhile, durability is associated to the fact that a product becomes spoilt, unsavory or past the best-before or use-by date.

### 2.4. Socio-economic characteristics and food-related behaviors

The socioeconomic characteristics and the food-related behavior are listed in Tables 1, 2. The socio-demographic variables considered were the type of household with children, the age and working status of the responsible for the purchase, the size of the household, and monthly household income. In particular, the responsible for purchase was asked to provide numerical values for the age, number of household members, and monthly household income. Meanwhile, a multiple-choice question was applied to collect the data related to the type of household and the working status (see Table 3).

**Table 1:** Socio-demographic characteristics of the sample analyzed in Cerdanyola del Valles.

Socio-economic factors	Variable	Missing value	No. Observation	Frequency
Gender	Male	2	242	30%
	Female		562	70%
Age	<54 years old	0	636	79%
	≥54 years old		170	21%
Work status	Employed	0	630	78%
	Not employed		176	22%
Household size	<4 people	0	380	47%
	≥4 people		423	53%
Household income	Low income	94	116	16%
	Medium low-income		194	27%
	Medium high-income		172	24%
	High-income		230	32%
Household food expenditure	<500 Euro/month	132	410	61%
	>500 Euro/month		264	39%

**Table 2:** Food-related behaviors of the household with children analyzed in Cerdanyola del Valles.

Food-related behavior factors	Variable	No. Observation	Frequency
Market channel	Supermarket	418	52%
	Other: grocery store, municipal market, alternative market	388	48%
How do you organize your food shopping?	I buy the majority of food in a single purchase+supplement shopping	574	71%
	I buy the majority of my food at different times throughout the week	232	29%
Do you prepare a shopping list before purchasing?	Yes	556	69%
	No	250	31%
How do you organize the food preparation at home?	I cook every day	126	16%
	I do not cook every day	680	84%
How do you define your diet?	Mainly animal-based(animal product consumption >4 days per week)	774	96%
	Mainly plant-based (animal product consumption <4 days per week)	31	4%
Frequency of fresh food consumption at home	Daily	532	66%
	Not daily	274	34%

**Table 3:** Socio-economic and food-related behaviors identifies as predictors for food waste generation in the household with children.

Variable	Odd ration	Confidential level 95%		Pr > Chi^2^	LR Chisq
		Lower bound	Upper bound		
Age of the responsible of purchase	1.0	1.0	1.1	<0.001	6.6
Household with young children					20.7
Household with middle-age children	1.0	0.6	1.6	0.83	
Household with adult children	2.2	1.2	4.2	>0.01	
Single-parents household	2.8	1.5	5.5	>0.001	
Main purchase channel: Supermarket					6.1
Main channel: no supermarket	1.6	1.1	2.3	>0.05	
Realize shopping list (No)					7.6
Realize shopping list(Yes)	1.8	1.2	2.8	>0.05	

The probability of declared food waste is expressed in odds ratio with the upper and lower bound to achieve a confidence level of 95%.

Regarding food purchase and preparation behavior, the questionnaire includes questions related to the main purchasing channel, the frequency and organization of food purchases, and the frequency of food preparation. The main purchasing channel was identified among supermarkets, grocery shops, and alternative markets; meanwhile, the frequency of purchase was assessed with a multiple-choice question (answer format: “I buy the majority of food in a single purchase+supplement shopping” and “I buy the majority of my food at different times throughout the week”). The organization of the purchase was assessed with yes or no questions related to the realization of a shopping list. Furthermore, the preparation behavior was identified based on the number of times the household prepares food at home during a week (answer format: four-point scale ranging from one time per week to daily). Finally, the diet quality at home was assessed with either the frequency of fresh food consumption (Seven-point scale ranging from once a fortnight to never to daily) and the type of diet through a multiple-choice question (answer format:” Mainly animal-based, animal product consumption >4 days per week” and “Mainly plant-based (animal product consumption <4 days per week”).

### 2.5. Data analysis

The statistical analysis for this study was performed using the latest available version of R software (R-4.3.0). In particular, the analysis was divided into two subsequent steps such as (1) the descriptive statistics to summarize the data and (2) the binary logistic regression to assess the variable’s capacity to predict food waste generation.

First, the socioeconomic and food-related behaviors were analyzed and summarized. Then the waste generated during the last seven days, the number of wasted products, product group, and the disposal reason were examined regarding the household life cycle stage. The analysis at household level has a specific interest regarding potential policy and prevention measures. Then, the relationship between food waste behavior and the socio-demographic variable and food behavior was assessed though a binary logistic regression. A dummy variable called “Declared food waste” was used as a dependent variable in the binary logistic regression, and the 14 variables were used as explanatory variables. After the first round of analysis, the explanatory variables that were not significant were excluded, and a second regression was performed with the remaining variables. A value of p of less than 0.05 was considered statistically significant. Multicollinearity was checked using Pearson correlation to determine the correlation between independent variables. The outliers have been examined and removed from the numerical variables such as age and household monthly purchase. The results were presented with an interval corresponding to a confidence level of 95%. ANOVA analysis was performed to assess the relative importance of the variable on predicting food waste generation. Furthermore, the accuracy test was conducted to evaluate the predictive performance of the logistic regression model. Specifically, the proportion of correct predictions over 500 observations obtained from the same model was assessed. As a fundamental diagnostic measure for logistic regression, the accuracy test provides valuable insights into the model’s predictive accuracy.

## 3. Results

Table 1 provides an overview of the demographic composition of the sample analyzed in this study. The majority of responsible individuals for food purchases were female (70%), under the age of 54 (79%), and employed part-time or full-time (78%). Approximately half of the families with children included in this study comprised less than four members (Mean: 3.57). Regarding household income, 16% reported a monthly income below 1.500 Euros, while 32% reported a monthly income greater than 3.500 Euros. Given that Catalonia’s average net household income is 3,000 Euros per month (29), families reporting a *monthly income* below 1,500 Euros were defined as “low-income households.,” between 1.500 Euros and 2.000 Euros were defined as “medium low-income household,” between 2.500 Euros and 3.500 Euros were defined as “medium high-income” and higher than 3.500 Euros “high-income household.” Among the families with children in the study, 61% reported spending less than 500 Euros per month on food and non-alcoholic beverages, however several outliers were identified (mean: 537 Euros/month). This value is higher than the regional statistics reported for Catalonia, which indicates an average monthly expenditure of 450 Euros on food and non-alcoholic beverages in families with children (34). The difference in reported expenditures may be attributed to the difficulty responsible individuals face in accurately estimating the amount spent on food and beverages each month. This difficulty is supported by the fact that approximately 15% of families could not respond to the expenditure question and the presence of several outliers in the analysis.

Table 2 provides an overview of the food-related behaviors reported by families with children. Consistent with regional food consumption trends, half of the households purchase groceries from supermarkets/hypermarkets (35). Two out of three families make a single shopping trip and supplement it with smaller purchases throughout the week, while one out of three families only shop at one location. Moreover, 70% of families reported making a grocery list based on what is needed at home before heading to the store. Regarding food consumption behavior, 66% of families reported consuming fresh products at home daily, while almost all families consume animal products at home more than four days a week.

### 3.1. Food waste generation in the household with children

**Figure 1:**
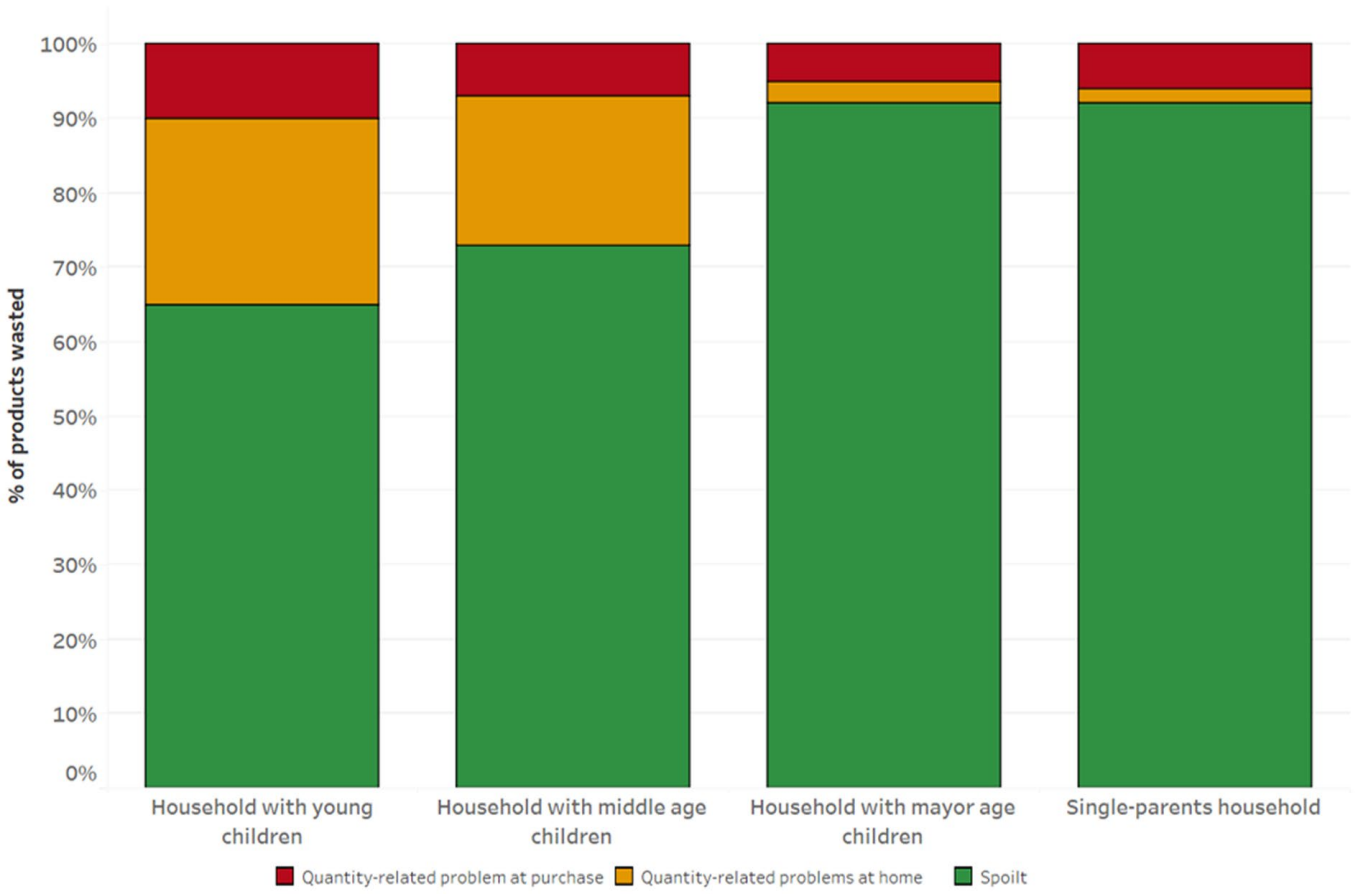
Product categories wasted in the different types of household with children.

During the seven days analyzed through a questionnaire, 63% of households with children reported disposing of less than two food items. Families with young (3.2) and middle-aged (2.4) children had the highest average number of discarded products, while single-parent families (1.8) and families with adult children (1.5) reported the lowest values. The food items most frequently discarded belonged to the category of vegetables (80%), followed by fruit (78%) and cereal-based product (63%). Dairy products also represented a frequently discarded product group, with 25% of families reporting throwing them away particularly, the household with young reported the highest percentage of dairy products wasted during the analysis (Figure 1). Meanwhile, animal-derived products such as meat, fish, and eggs were the least frequently discarded, along with ready-to-prepare/eat products.

**Figure 2:**
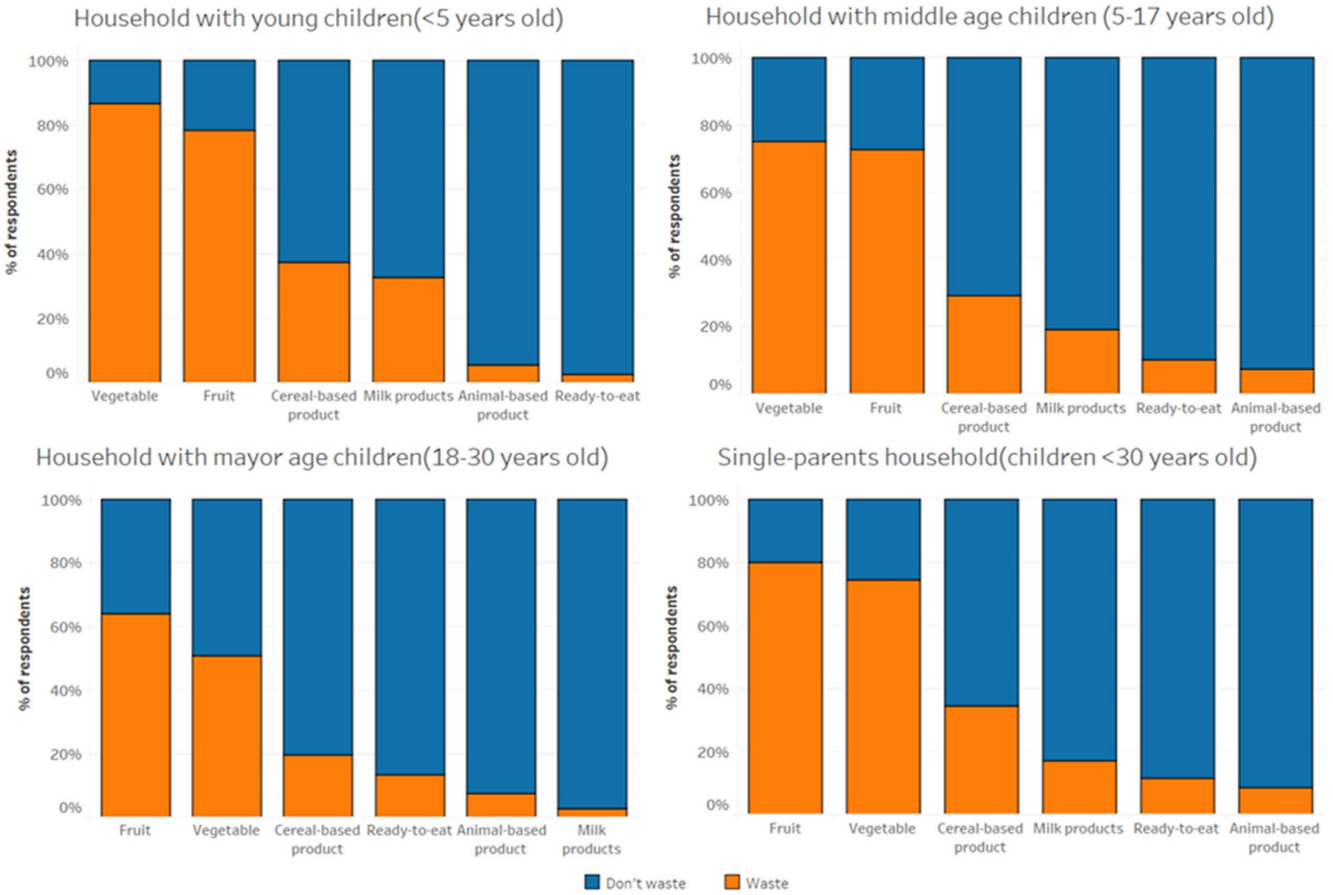
Reasons for disposal are listed for the different type of the households with children. The reasons for disposal included in the analysis are the quantity-problems at purchase, home and spoilt.

Regarding the reasons for disposal, two out of three families reported throwing away most of their food items without having used them. The vast majority of the food were wasted due to the durability of the product (76%), while only a tiny portion of the products were discarded due to quantity problems related at purchase (10%) or at home (14%). In Figure 2, it can be observed that families with young and middle-aged children reported the highest levels of food waste due to quantity-related issues. In these families, quantity problems were mainly related to preparing too much food, and to a lesser extent, to over-purchasing. Conversely, most of the families with adult children and single-parent households wasted products due to durability. Furthermore, the causes of waste due to quantity were primarily related to over-purchasing, which was often attributed to purchasing products in excessively large packaging sizes.

### 3.2. Regression results

The last data analysis step was estimating the binary regression models to investigate the drivers generating domestic food waste in households with children. The regression model included six socioeconomic factors such as the sex of the responsible for purchase (reference value: male), age of the responsible for purchase (from smallest to largest, numerical value), working status of the responsible for purchase (reference value: employed), type of household (reference: household with young children), household size (from smallest to largest, numerical value), annual household income (reference value: <2.500 Euro per month) and household expenditure in food (from smallest to largest, numerical value). In addition, the food-related behavior included six factors in the regression model as the primary purchase channel (reference value: supermarket/hypermarket), frequency of purchase (reference value: one general purchase per week + complimentary shopping), elaboration of a shopping list (reference value: yes), frequency of food preparation (reference: daily), the type of diet (reference value: mainly animal-based(animal product consumption >4 days per week) and the frequency of fresh product consumption (reference value: daily).

The logistic regression model successfully identified four predictors of food waste generation in households with children. Table 3 illustrates each factor’s variables, degree of significance, odds ratio at the 95% confidence level, and the results of the ANOVA analysis. After the first round of the binary regression model, four factors with statistically significant parameters were retained and inputted in the final regression model. Table 2 illustrates each factor’s variables, degree of significance, odds ratio at the 95% confidence level, and the results of the ANOVA analysis. No factors were excluded due to multicollinearity during the analysis. The outlier analysis excluded 20% of the data concerning monthly household expenditure, while no outliers were identified for the age of the household head. For further details on the results of the first regression model see Supplementary Table S2 in the Supplementary Material.

Among the socioeconomic variables examined, the age of the household head and family type were significant predictors of food waste generation. The analysis revealed a negative relationship between the age of the household head and the likelihood of food waste generation, indicating that older household heads were associated with a lower probability of generating food waste (odds ratio = 1.04, *p* < 0.01). In terms of family type, households with adult children (odds ratio = 2.27, *p* < 0.05) and single-parent families (odds ratio = 2.8, *p* < 0.001) were found to have a lower probability of wasting food compared to households with small and medium-sized children. Regarding food-related behaviors, households that reported procuring their food from grocery stores and municipal markets had a lower probability of generating food waste than those who obtained their food from the supermarket/hypermarket channel (odds ratio = 1.54, *p* < 0.05). Moreover, a lower probability of food waste was associated with creating a shopping list before grocery shopping (odds ratio = 1.82, p < 0.05).

The ANOVA analysis highlighted that the household type (LR Chisq = 20.7) and the realization of the shopping list before purchase (LR Chisq = 7.6) are the factors with the higher impact on the variation in the prediction of food waste generation. On the other hand, the age responsible for the purchase (LR Chisq = 6.6) and the purchase channel for groceries (LR Chisq = 6.1) have a lower impact on the prediction.

## 4. Discussion

### 4.1. Food waste characterization in the household with children

The data set showed that 31% of the households did not record food waste during the previous seven days. Related information from food waste studies realized in Spain, Italy, and Denmark suggest that between 15 and 40% of respondents to questionnaires stated not wasting any edible food within a regular week or during the previous week (27, 28, 35). As self-reported food waste is prone to social desirability and memory bias (33), the request to declare whether the household wastes some edible product instead of quantifying we attempted to minimize this bias. Indeed, several studies found self-reported food waste highly subjected to underreporting (13, 36, 37), especially in households with multiple members (38). Despite our efforts, the findings of our study are influenced by subjective perceptions of edibility, particularly when determining whether certain parts of a food item, such as vegetable peels, are edible or not. This makes it challenging to categorize food products as either edible or inedible in advance. However, unlike other studies that rely on questionnaires to measure household food waste, we aimed to gain a more nuanced understanding of households with children prone to generating food waste.

Unsurprisingly perishable food, such as fruits, vegetables, and cereal-based product, are the primary items discarded, as previous studies have shown (13, 39). In addition, our findings indicate that dairy product contribute significantly to domestic food waste in households with young children and middle-aged individuals. This result highlights the importance of not excluding a specific dairy product, such as milk, from food waste quantification studies in households with children, as it is a common practice to exclude liquids in current literature (28, 40). Respondents reported spoilage as the primary reason for food waste in their homes, which aligns with other literature (14, 36).

Previous research by Herzberg et al. (13), Koivopuro et al. (41), and Falasconi et al. (27) has indicated that households with young and middle-aged children exhibit a higher prevalence of quantity-related issues in the context of serving or preparing excessive food compared to other family types. Such problems may be attributed to these households’ challenges in providing healthy and appropriate food for their children (20, 42, 43) while also managing their children’s finicky eating habits (15). The analysis confirmed the higher presence of quantity-related problems in families with young and middle-aged children. Additionally, it was highlighted that households with older children and single-parent households reported the most significant waste causes due to spoilt and quantity-related problems during purchase. This evidence suggests the presence of two distinct patterns of food waste in the household with children. Specifically, households with young and middle-aged children appear to waste food due to inadequate food management strategies, resulting in frequent small amounts of waste. In contrast, food waste in other households with children, and more in general to the other type of households, may result from over-purchasing perishable items discarded before consumption.

### 4.2. Prediction of food waste generation through socio-demographic and food-related behavior variables

Binary logistic regression identified four variables to identify which households and behaviors should be prioritized for food waste reduction. Household type and making a shopping list before going shopping are the two most influential factors in the model studied. The higher likelihood of food waste in families with young and middle-aged children empirically confirms the child’s age as a valid variable for predicting food waste generation, consistent with the qualitative analysis of Kansal et al. (18). The greater time and money constraints may underlie this higher likelihood of food waste, as indicated by Parizeau et al. (14). Meanwhile, careful grocery shopping planning has been confirmed as a critical behavior to reduce food waste at home, even for families with children. Many studies have stated that careful grocery shopping planning is an effective tool to prevent overbuying and, consequently, food waste (14, 44, 45). Moreover, the limited use of the shopping list could serve as a proxy for other planning behaviors, which might explain the increased occurrence of food waste (46). Indeed, Quested et al. (37) have shown a robust positive association between the creation of shopping lists and “planning behavior,” such as the premeditated planning of meals, inspection of existing food inventories before shopping, employment of freezing techniques to prolong the shelf life of food, and repurposing of leftovers.

In addition to the type of household and the purchase planning, the buyer’s age and the purchasing channel were found to be factors with predictive power within the model. The inverse relationship between the age of the person in charge of food purchasing and the probability of generating food waste is consistent with previous studies (13, 36, 47). As well as other studies have also reported a higher likelihood of waste among individuals who purchase groceries from supermarkets (48–50). Specifically, products in predetermined packaging or products on discount (i.e., 3×2) induce consumers to buy more food than necessary for their families. In addition to the method of selling products, the presence of ultra-processed food items such as unhealthy snacks, frozen pizza, ice cream, and flavored yogurt in supermarkets has been indicated as a reason for food waste generation at home. Indeed, consuming these products, particularly appreciated by adolescents and young children (51), can compete with healthy product products such as fruits and vegetables served during mealtimes. Graham-Rowe et al. (20) showed that some parents frequently over-portion dinner for children to discourage eating unhealthy snacks, which could lead to significant food waste. Moreover, supermarkets are known for their persuasive marketing appeals targeting children. Chen et al. (52) and Haselhoff et al. (53) highlight that children can employ persuasion, begging, and emotional appeals to influence their parents into purchasing products with attractive packaging or advertised on television.

In contrast to several studies, household income (36, 47, 54, 55) and the number of household members (56–58) were not found to be predicting factors for food waste generation in families with children. The focus of this study on a specific segment of the population characterized by a similar number of household members and monthly income levels explains this finding. This result puts into perspective the importance of these two factors as predictors of food waste within the population, given that families with children typically have higher incomes and a more significant number of household members. However, the fact that the analysis does not quantify food waste but only assesses whether or not waste was generated in the last week may explain why these two variables were not significant.

Regarding the methodology used in this study, the application of predictive models in food waste research during the consumption phase is increasing during the last decade (59). Previous research has demonstrated how predictive models can be customized for various sectors with limited utilization of these statistical techniques (60, 61). The food waste behavior was analyzed both with parametric (17, 62) and no parametric machine learning algorithm (63, 64). The choice to employ a parametric model for this research is based on two primary factors. Firstly, the analyzed variables have been observed in numerous studies, emphasizing the need for a well-established and interpretable approach. Secondly, the main objective of this analysis is to achieve accurate predictions rather than exploring intricate relationships or uncovering hidden patterns within the data. Additionally, focusing on a specific segment of the population helps mitigate the presence of outliers within the sample, while the ample sample size enables the model to be less susceptible to overfitting. Parametric models using both linear regressions (10, 13, 62) and logistic regression (17, 27, 28). These two predictive models differ in their output and data requirements. Logistic regression, utilizing a dummy variable, determines whether a household wastes food but does not provide information on the extent of the food waste. However, challenges and costs associated with quantitative analyses of consumption-related food waste, along with uncertainties due to cognitive biases and different perceptions of food waste among users (65, 66), limit the scalability of the predictive linear regression model. The use of categorical variables to assess food waste behavior (i.e., Declared food waste) enables quick and cost-effective data collection through questionnaires giving the possibility to increase the number of samples analyzed. The increase in the number of studies on food waste is fundamental for deepening knowledge about food waste and developing strategies to decrease it.

### 4.3. Policy implications

This section presents policy implications for addressing food waste in households with children based on the findings of this research and evidence from the literature. As highlighted by the analysis, policies aimed at preventing food waste should focus on young families with small and medium-sized children. Specifically, policy actions are required to facilitate changes in the perception of waste, promote good practices, and create favorable conditions for reducing food waste in these families. Placing the child at the center of policies to raise awareness about food waste is crucial, given their ability to directly and indirectly, influence waste (18). In this regard, promoting a healthy diet and describing the impact of food waste in schools can help reduce waste in families in the short term and create greater awareness of food waste in future families in the long term (15). As well as promoting local and seasonal products and menus mainly based on plants within schools can play a crucial role in educating children and supporting families in this critical aspect of daily life (67). Regarding this aspect, Šimanskienė et al. (68) recommended that educational institutions design lessons or lectures centered around responsible consumption topics. In particular, the development of methodological resources, such as exercise books and the creation of computer games that demonstrate the impact of individual consumption and behavior, can help educate students about responsible consumption and its role in environmental preservation.

Concerning good practices, the policies should stimulate planning behavior within the household with children, such as creating a shopping list, pre-planning meals and inspecting existing food stocks before purchasing (37). These behaviors can be stimulated through the promotion of online tools and apps for creating shopping lists (69), managing existing food stocks (70). Given the significance of food waste related to quantity problems arising from over-preparation of food, it would be beneficial to assist households in reusing leftoversby providing recipes particularly for perishable product. This can be achieved by promoting food reusing apps [e.g., (71)], launching campaigns [e.g., (72)], and incorporating them into product packaging. Moreover, enlisting children’s participation in activities such as meal planning and food preparation, such as cooking together, are practices that have been found to have a constructive impact on dietary patterns and food waste generation (18).

Public policies should strive to create favorable conditions to reduce food waste in households with young and middle age children. Firstly, policies should encourage responsible consumption within the population through awareness-raising campaigns (73). In particular, parents should be informed as much as possible about the food waste impact on the environment and the region in which they live. Concerns about food waste and moral attitudes (i.e., feelings of guilt when discarding food) are crucial to determine their intention not to waste food and reshape the individual attitude toward buying and consuming food (74). For example, positive correlation between the variables planning routines and awareness of environmental problems has been observed by Fiore et al. (75). Secondly, specific policies are required to incentivize the sale of bulk products and limit the promotion of ultra-processed products in the supermarket. Focusing on supermarkets is necessary since most people use this purchasing channel as the main one due to time and money constraints. In this sense, promoting the sale of bulk products helps the shopper buy the exact amount of products needed for the household by limiting overbuying and reducing the generation of other packaging waste. Furthermore, developing customized packaging for products such as fruits and vegetables targeted toward families with children could partially alleviate quantity-related problems at purchase (i.e., less quantity and more variety). On the other hand, to reduce the consumption and potential distractions caused by ultra-processed products when purchasing goods, limiting their promotion campaigns aimed at adolescents and young children is crucial. One strategy to achieve this is placing these products in less visible locations, such as on top shelves or in areas the responsible shopper may not pass by.

Finally, it is essential to mention that policies for managing organic waste should be implemented in addition to food waste prevention policies. Food waste management accounts for approximately one-third of the entire food system’s greenhouse gas (GHG) emissions (76) due to the widespread use of landfilling and open dumping, both of which are strongly linked to high GHG emissions (77). By implementing the separate collections in the neighborhoods with a high percentage of households with children, the potential to employ low-carbon-footprint waste management technologies such as aerobic digestion and composting is increased, which can help to mitigate GHG emissions. This two-pronged approach of preventing and managing organic waste is essential to decrease food waste and its impact on families with children in parallel.

## 5. Conclusion

The issue of food waste is a significant challenge faced by societies worldwide. This study adds to the growing body of literature on household food waste by examining the food waste behaviors of households with children and identifying factors that can predict food waste generation. Our results show that perishable food items such as fruits, vegetables, bread, and dairy products are the primary items discarded. In particular, quantification analyses of food waste in households with children should take into account liquid waste, especially from milk, to avoid underestimating the problem. The study also identified two patterns of food waste in households with children: inadequate food management strategies resulting in frequent small amounts of waste in families with young and middle-aged children, and over-purchasing perishable items discarded before consumption in other households with children.

These findings are crucial for policymakers and stakeholders as they can help in developing targeted interventions to reduce household food waste, particularly for families with young and middle-aged children. Based on our study, strategies to reduce food waste in households with children should focus on improving food management strategies and promoting planning behavior such as making shopping lists. Schools can play a vital role in educating children and supporting families, while online tools and apps can encourage good practices such as planning and reusing leftovers. Policies that incentivize the sale of bulk products and limit the promotion of ultra-processed items in supermarkets can also be implemented, along with policies for managing organic waste to decrease their impact on families with children. It is important to note that reducing household food waste requires a multi-stakeholder approach involving policymakers, food manufacturers, retailers, and consumers. The results of this study contribute to the growing body of literature on household food waste and emphasize the importance of considering socio-demographic and food-related behavior variables in predicting food waste generation and implementing effective interventions.

Future research using a predictive model for food waste behavior should focus on young people living alone or sharing a house, identified by different studies as the household type generating the highest kg *per capita* of food waste (13, 16, 41). As well as in comparing different populations with different levels of awareness on food waste aspects or shed light on the cultural impact of food waste. Finally, to advance in the study of food waste in households with children, research should focus on assessing which variable identified by logistic regression has the most significant weight. In this case, quantifying food waste in a small sample would make it possible to define which behavior should be prioritized in a given population segment.

## Data availability statement

The raw data supporting the conclusions of this article will be made available by the authors, without undue reservation.

## Author contributions

PT: Conceptualization, Data curation. XD, PO and PT: Formal Analysis. PT: Methodology. XD: Funding acquisition. XD: Project administration. PT: Roles/Writing — original draft. XD and PO: Writing — review & editing. All authors contributed to the article and approved the submitted version.
