# Supplementary material for: Predicting food waste in households with children: socio-economic and food-related behavior factors
Source: Front Nutr. 2023 Oct 9;10:1249310. doi: 10.3389/fnut.2023.1249310 (PMC10591224; doi:10.3389/fnut.2023.1249310)
Supplement: Supplementary file 1 [file Table_1.DOCX]

**Supplementary Material**

| Lifecycle Stages | Further Specifications |
| --- | --- |
| Families with young children | Youngest child <5 years old |
| Families with middle-age children | Youngest child between 5-18 years old |
| Families with mayor age children | Youngest child between 18-30 years old |
| Single-parents household | Mother or father, youngest children< 30 years old |

Table S1. Detailed specifications of the socio-demographic characteristic lifecycle stage.

| Variable | Odd Ration | Confidential Level 95% | | Pr > Chi^2^ | LR Chisq |
| --- | --- | --- | --- | --- | --- |
|  |  | Lower Bound | Upper Bound |  |  |
| Male |  |  |  |  | 3.3 |
| Female | 0.7 | 0.4 | 1.0 | 0.1 |  |
| Employed |  |  |  |  | 2.3 |
| Not employed | 0.7 | 0.4 | 1.1 | 0.1 |  |
| Household with young children |  |  |  |  | 13.1 |
| Household with middle-age children | 1.0 | 0.6 | 1.8 | 0.98 |  |
| Household with adult children | 2.1 | 1.0 | 4.4 | >0.05 |  |
| Single-parents household | 3.0 | 1.4 | 6.4 | >0.01 |  |
| Household size | 1.0 | 0.7 | 1.3 | >0.01 | 0.02 |
| Low income |  |  |  |  | 1.1 |
| Medium-low income | 1.0 | 0.5 | 1.8 | 1.0 |  |
| Medium-high income | 0.9 | 0.5 | 1.9 | 0.8 |  |
| High income | 0.7 | 0.4 | 1.5 | 0.4 |  |
| Household food expenditure | 1.0 | 1.0 | 1.0 | 0.5 | 0.4 |
| Main purchase channel: Supermarket |  |  |  |  | 10.6 |
| Main channel: no supermarket | 2.0 | 1.3 | 3.0 | >0.01 |  |
| I buy the majority of food in a single purchase+supplement shopping |  |  |  |  | 1.5 |
| I buy the majority of my food at different times throughout the week | 0.7 | 0.5 | 1.2 | 0.2 |  |
| Realize shopping list (No) |  |  |  |  | 6.1 |
| Realize shopping list(Yes) | 1.9 | 1.1 | 3.1 | >0.05 |  |
| I cook every day |  |  |  |  | 0.8 |
| I don't cook every day | 1.1 | 0.6 | 1.9 | 0.8 |  |
| Frequency of fresh food consumption at home:Daily |  |  |  |  | 0.2 |
| Frequency of fresh food consumption at home: Not daily | 1.1 | 0.7 | 1.7 | 0.7 |  |
| Mainly animal-based( animal product consumption >4 days per week) |  |  |  |  | 4.1 |
| Mainly plant-based (animal product consumption <4 days per week) | 0.3 | 0.1 | 1.0 | 0.1 |  |

Table S2. Socio-economic and food-related behaviours factor analysed in the first model before the exclusion of the no-significant factors.The probability of declared food waste is expressed in odds ratio with the upper and lower bound to achieve a confidence level of 95%.
